# Multiple Laplacian Regularized RBF Neural Network for Assessing Dry Weight of Patients With End-Stage Renal Disease

**DOI:** 10.3389/fphys.2021.790086

**Published:** 2021-12-13

**Authors:** Xiaoyi Guo, Wei Zhou, Yan Yu, Yinghua Cai, Yuan Zhang, Aiyan Du, Qun Lu, Yijie Ding, Chao Li

**Affiliations:** ^1^Hemodialysis Center, The Affiliated Wuxi People’s Hospital of Nanjing Medical University, Wuxi, China; ^2^Institute of Fundamental and Frontier Sciences, University of Electronic Science and Technology of China, Chengdu, China; ^3^Department of Nursing, The Affiliated Wuxi People’s Hospital of Nanjing Medical University, Wuxi, China; ^4^Yangtze Delta Region Institute (Quzhou), University of Electronic Science and Technology of China, Quzhou, China; ^5^General Hospital of Heilongjiang Province Land Reclamation Bureau, Harbin, China

**Keywords:** end-stage renal disease, dry weight, RBF networks, multiple Laplacian regularized model, machine learning

## Abstract

Dry weight (DW) is an important dialysis index for patients with end-stage renal disease. It can guide clinical hemodialysis. Brain natriuretic peptide, chest computed tomography image, ultrasound, and bioelectrical impedance analysis are key indicators (multisource information) for assessing DW. By these approaches, a trial-and-error method (traditional measurement method) is employed to assess DW. The assessment of clinician is time-consuming. In this study, we developed a method based on artificial intelligence technology to estimate patient DW. Based on the conventional radial basis function neural (RBFN) network, we propose a multiple Laplacian-regularized RBFN (MLapRBFN) model to predict DW of patient. Compared with other model and body composition monitor, our method achieves the lowest value (1.3226) of root mean square error. In Bland-Altman analysis of MLapRBFN, the number of out agreement interval is least (17 samples). MLapRBFN integrates multiple Laplace regularization terms, and employs an efficient iterative algorithm to solve the model. The ratio of out agreement interval is 3.57%, which is lower than 5%. Therefore, our method can be tentatively applied for clinical evaluation of DW in hemodialysis patients.

## Introduction

Dry weight (DW) refers to a patient’s target weight after the end of dialysis (Grassmann et al., 2000; Wabel et al., 2009). After removing excess water from the body, the patient had no facial swelling, wheezing or sitting breathing, edema of both lower limbs, and distended jugular vein (Alexiadis et al., 2016). The patient’s blood pressure, heart rate, breathing, and other vital signs are stable. There are individual differences in the specific value of DW. Good dry weight control can effectively reduce adverse reactions during dialysis. At present, the DW of hemodialysis patients is mainly evaluated by clinical means. This method is labor-intensive and time-consuming, and requires repeated use of various clinical instruments and biological indicators to complete the evaluation. In the past 10 years, a measuring instrument based on human bioelectrical impedance analysis (BIA), called body composition monitor (BCM) (Jiang et al., 2017), has been accurately determining the DW of patients. The above methods require professionals and cannot be processed on a large scale.

In recent years, artificial intelligence technology has been widely utilized in the biomedical field (Chen et al., 2019; Cheng et al., 2019; Lin, 2020; Zhang, 2020; Hu et al., 2021a). Artificial neural networks (ANNs) based on back propagation (BP) were employed to evaluate the total water volume of hemodialysis patients. Compared with conventional clinical calculation equations (Chiu et al., 2005), the ANNs obtained better results. Deep learning (Dao et al., 2021; Lv et al., 2021a,b) also made a great contribution to the clinic, including skin cancer (Esteva et al., 2017), breast cancer (Liu J. et al., 2021), and brain diseases (Liu G. et al., 2018; Liu et al., 2019; Bi et al., 2020; Hu et al., 2020, 2021a,b). In biological field, machine learning has been widely used to solve biological problems, including *O*-GlcNAcylation site prediction (Jia et al., 2018), microbiology analysis (Qu et al., 2019), microRNAs and cancer association prediction (Zeng et al., 2018), lncRNAs (Cheng et al., 2016; Deng et al., 2021), CircRNAs (Fang et al., 2019; Zhao et al., 2019), DNA methylation site (Wei et al., 2018b; Zou et al., 2019; Dai et al., 2020), osteoporosis diagnoses (Su et al., 2020b), function prediction of proteins (Wei et al., 2018a; Wang H. et al., 2019; Deng et al., 2020b; Ding et al., 2020a; Su et al., 2020a), nucleotide binding sites (Ding et al., 2021b), drug complex network analysis (Ding et al., 2019, 2020b,a; Deng et al., 2020a; Han et al., 2021; Liu H. et al., 2021), protein remote homology (Liu B. et al., 2018), electron transport proteins (Ru et al., 2019), and cell-specific replication.

In this study, we proposed a novel predictive model based on radial basis function neural network (RBFN). Different from RBFN, multiple Laplacian-regularized RBFN (MLapRBFN) is a multi-view Laplacian regularized model with L_2_,_1_-norm, which introduces multiple graph regular items. The Laplacian regular items consider the topological relationship between each patient.

## Materials and Methods

### Materials

The data set of this study came from the hemodialysis center of Wuxi and the northern Jiangsu People’s Hospital. Our study was approved by the ethics committees (Nos. 2018KY-001 and KYLLKS201813). There are a total of 476 hemodialysis patients. These patients meet the following conditions: over 18 years old; more than 3 months end-stage renal disease (ESRD) and maintenance hemodialysis; diseases such as metal implants, infections, heart failure, disability, pregnancy, and edema, do not appear in the patient population. DW is determined by clinical scoring, which is based on brain natriuretic peptide (BNP), chest computed tomography (CT) image, ultrasound, and bioelectrical impedance analysis (BIA). In addition, age, gender, diastolic blood pressure (DBP), systolic blood pressure (SBP), years of dialysis (YD), heart rate (HR), and body mass index (BMI) are employed to construct our model. The summary of information is shown in Table 1. This study is a retrospective study, and feature of patient is easy to obtain. We hope to provide patients with a non-invasive dry weight assessment method through machine learning models.

**TABLE 1:** She summary information of patients.

Feature	*Value*	*r* *
Gender (males/females)	312/164	–0.4489
Age (years)	54.17 ± 14.22	–0.2341
BMI	22.96 ± 2.95	0.9558
HR (times/min)	73.41 ± 8.92	0.1862
DBP (mmHg)	88.32 ± 19.56	–0.1249
SBP (mmHg)	150.64 ± 29.36	–0.1739
YD (years)	5.97 ± 3.22	–0.1069

*Denotes correlation coefficient between individual variables and dry weight value.

### Radial Basis Function Network

**FIGURE 1:**
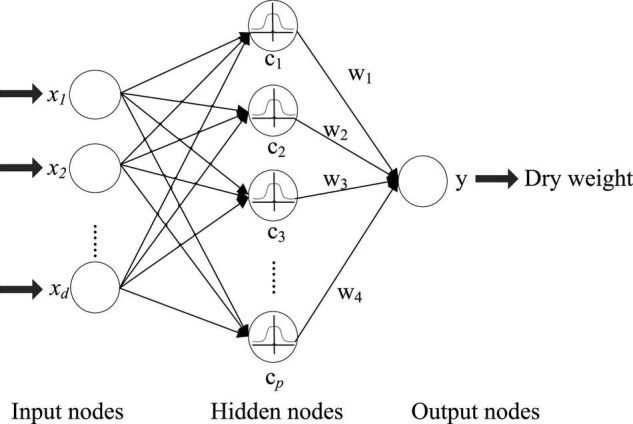
Topological structure of the radial basis function (RBF) network.

The RBF neural network (Park and Sandberg, 1990) is composed of an input layer, a hidden layer, and an output layer, and is shown in Figure 1. The feature of the hemodialysis patient can be fed into the RBF network for processing. Suppose that there are *N* samples containing *d* variables ({*x*_*i*_, *y*_*i*_}, *i* = 1, 2, …, *N*). The output is *y*_*i*_ ∈ *R*^1×*q*^ and input data *x*_*i*_ ∈ *R*^1×*d*^. In Figure 1, the RBF network has three layers, which are the input, hidden, and output layers. The transformation from the input space to the hidden layer space is non-linear, and the transformation between the hidden layer and the output layer is linear. The fundamental of the RBFN is the RBF is the “base” of the hidden unit. The vector of input layer can be mapped to the space of the hidden layer without weight connection. The mapping relationship is determined with center point of hidden unit. In Figure 1, the number of hidden layer nodes is *p* (*p* center points). The activation function of the RBF neural network can be represented as:


(1) $$\phi \left(x_{i},c_{j}\right)=\exp\left(-\frac{1}{2}\sigma^{2}{||x_{i}-c_{j}||}^{2}\right)$$

where *x*_*i*_ is a feature vector of sample, *c*_*j*_, *j* ∈ {1, 2, …, *p*} is the vector of *j*-th center point, and σ is the width parameter of the function, which controls the radial range of the function. RBFN can be represented as:


(2) ΦW=Y

where matrix Φ ∈ *R*^*N*×*p*^ is the output of the hidden nodes. *W* ∈ *R*^*p*×*q*^ is the weight matrix between output and hidden nodes. *Y* ∈ *R*^*N*×*q*^ is the matrix of dependent variable. Φ ∈ *R*^*N*×*p*^ and *W* ∈ *R*^*p*×*q*^ can be represented as:


(3) $$\Phi ={\left[\begin{array}{ccc} \phi \left(x_{1},c_{1}\right) & \cdots & \phi \left(x_{1},c_{p}\right)\\ \vdots & \ddots & \vdots \\ \phi \left(x_{N},c_{1}\right) & \cdots & \phi \left(x_{N},c_{p}\right) \end{array}\right]}_{N\times p}$$

(4) $$W={\left[\begin{array}{c} w_{1}^{T}\\ w_{2}^{T}\\ \vdots \\ w_{p}^{T} \end{array}\right]}_{p\times q}$$

To train RBFN, 3 parameters should be solved: the center points of the basis function (*c*_*j*_ ∈ *R*^1×*d*^, *j* ∈ {1, 2, …, *p*}), width (σ), and weight matrix between the output and hidden layer (*W* ∈ *R*^*p*×*q*^). In most cases, the self-organized selection center learning method is used: (1) unsupervised learning process, solving the center points and width of RBF, (2) supervised learning process, solving the weights *W* ∈ *R*^*p*×*q*^. First, select *p* centers for k-means clustering (*p* clusters). For the radial basis of the Gaussian kernel function, the width is solved by the formula:


(5) $$\sigma_{j}=\frac{\sigma_{\max}}{\sqrt{2p}},j=1,2,\ldots ,p$$

where σ_max_ is the maximum distance between the selected center points.

For *W* ∈ *R*^*p* × *q*^, the RBF network can be represented as:


(6) $$W^{*}=\arg\min\frac{1}{2}{||\Phi \text{W}-\text{Y}||}_{F}^{2}$$

The gradient of Eq. 6 can be set as 0:


(7) $$W^{*}={\left(\Phi^{\text{T}}\Phi \right)}^{-1}\Phi^{\text{T}}Y$$

For a new test sample *x*_*new*_, we can estimate $y_{new}^{*}$ as:


(8) $$y_{new}^{*}=\Phi_{new}W^{*}$$

where Φ_*new*_ = (*ϕ* (*x*_*new*_, *c*_1_), *ϕ* (*x*_*new*_, *c*_2_), …, *ϕ* (*x*_*new*_, *c*_*p*_))_1 × *p*_.

### Proposed Model of Multiple Laplacian-Regularized RBF Network

To further improve the generalization performance of the RBF network, a multi-view Laplacian-regularized RBF network is proposed. The unsupervised process of the first stage remains unchanged; we mainly improve the model in the second part. The objective function of Eq. 6 is revised as:


(9) $$\begin{array}{c} \underset{W,\eta_{v}}{argmin}\frac{1}{2}{||\Phi \text{W}-\text{Y}||}_{F}^{2}\\ +\frac{\lambda_{1}}{2}Tr\left({\left(\Phi W\right)}^{\text{T}}\sum_{v=1}^{V}{\left(\eta_{v}\right)}^{\rho }L_{v}\left(\Phi W\right)\right)\\ +\frac{\lambda_{2}}{2}{\left|W\right|}_{2,1}^{2}\\ s.t.\;\sum_{v=1}^{V}{\text{η}}_{v}=1,\\ 0<{\text{η}}_{v}<1,\\ v=1,2,\ldots ,V \end{array}$$

where *λ*_1_ and *λ*_2_ denote the parameters of Laplacian and L_2_, _1_-norm term. L_2_, _1_-norm can be used to obtain a sparser solution during the training process, which makes the model more robust. ρ > 1, which is used to prevent the extreme situations of η_*v*_ = 0 (or η_*v*_ = 1). *L*_*v*_ ∈ *R*^*N*×*N*^ is the Laplacian matrix, which is employed to represent the manifold of samples. *V* is the number of views. *L*_*v*_ is built by heat kernel matrix *S*_*v*_ ∈ *R*^*N*×*N*^:


(10a) $$L_{v}=D_{v}^{-1/2}\Delta_{v}D_{v}^{-1/2}$$

(10b) $$\Delta_{v}=D_{v}-S_{v}$$

(10c) $$D_{v}\left(i,i\right)=\sum_{j=1}^{N}S_{v}\left(i,i\right)$$

In our study, we employ the following functions to construct the heat kernel matrix:


(11a) $$S{\left(i,j\right)}_{1}=\exp\left(-\gamma {||x_{i}-x_{j}||}^{2}\right)$$

(11b) $$S{\left(i,j\right)}_{2}=\frac{x_{i}^{\text{T}}x_{j}}{\left|x_{i}\right|\left|x_{j}\right|}$$

(11c) $$S{\left(i,j\right)}_{3}={\left(-\gamma x_{i}^{\text{T}}x_{j}+0.1\right)}^{2}$$

(11d) $$S{\left(i,j\right)}_{4}=\tanh\left(-\gamma x_{i}^{\text{T}}x_{j}+0.1\right)$$

where γ is a constant, and we set it as 1.

### Optimization

The third term of ${||W||}_{2,1}^{2}$ cannot be diversified, so Eq. 9 is converted into:


(12) $$\begin{array}{c} \underset{W,\eta_{v}}{argmin}\frac{1}{2}{||\Phi \text{W-Y}||}_{F}^{2}\\ +\frac{\lambda_{1}}{2}Tr\left({\left(\Phi W\right)}^{\text{T}}\sum_{v=1}^{V}{\left(\eta_{v}\right)}^{\rho }L_{v}\left(\Phi W\right)\right)\\ +\frac{\lambda_{2}}{2}Tr\left(W^{\text{T}}GW\right)\\ s.t.\sum_{v=1}^{V}\eta_{v}=1,\\ 0<\eta_{v}<1,\\ v=1,2,\ldots ,V \end{array}$$

where *G* ∈ *R*^*p*×*p*^ is a diagonal matrix, and *i-*th is an element:


(13) $$G_{jj}=\frac{1}{2{||W_{j}||}_{2}},j=1,2,\ldots ,p$$

Since there are multiple variables (*W*, η_*v*_) that need to be optimized, we first fix η_*v*_ and optimize *W*. We initialize $\eta_{v}^{\left(0\right)}=1/V$, and get the fused matrix $L^{*\left(0\right)}=\sum_{v=1}^{V}\eta_{v}^{\left(0\right)}L_{v}$. Equation 12 can be written as:


(14) $$\begin{array}{c} \underset{W}{argmin}\frac{1}{2}{||\Phi \text{W-Y}||}_{F}^{2}\\ +\frac{\lambda_{1}}{2}Tr\left({\left(\Phi W\right)}^{\text{T}}L^{*}\left(\Phi W\right)\right)\\ +\frac{\lambda_{2}}{2}Tr\left(W^{\text{T}}GW\right) \end{array}$$

We obtain the derivative of formula (14) for variable *W*:


(15a) $$\Phi^{\text{T}}\left(\Phi \text{W-Y}\right)+\lambda_{1}\Phi^{\text{T}}L^{*}\left(\Phi W\right)+\lambda_{2}GW=0$$

(15b) $$\Phi^{\text{T}}\Phi W+\lambda_{1}\Phi^{\text{T}}L^{*}\Phi W+\lambda_{2}GW=\Phi^{\text{T}}Y$$

(15c) $$\left(\Phi^{\text{T}}\Phi +\lambda_{1}\Phi^{\text{T}}L^{*}\Phi +\lambda_{2}G\right)W=\Phi^{\text{T}}Y$$

(15d) $$W={\left(\Phi^{\text{T}}\Phi +\lambda_{1}\Phi^{\text{T}}L^{*}\Phi +\lambda_{2}G\right)}^{-1}\Phi^{\text{T}}Y$$

Then, we fix the variant *W* and optimize η_*v*_, *v* = 1, 2, …, *V*, which is related to:


(16) $$\begin{array}{c} \underset{\eta_{v}}{argmin}Tr\left({\left(\Phi W\right)}^{\text{T}}\sum_{v=1}^{V}{\left(\eta_{v}\right)}^{\rho }L_{v}\left(\Phi W\right)\right)\\ s.t.\;\sum_{v=1}^{V}\eta_{v}=1,\\ 0<\eta_{v}<1,\\ v=1,2,\ldots ,V \end{array}$$

The above formula can be converted to a Lagrange function:


(17) $$Lag\left(\eta_{v},\text{ξ}\right)=Tr\left({\left(\Phi W\right)}^{\text{T}}\sum_{v=1}^{V}{\left(\eta_{v}\right)}^{\rho }L_{v}\left(\Phi W\right)\right)-\text{ξ}\left(\sum_{v=1}^{V}\eta_{v}-1\right)$$

We set η_*v*_ and ξ to 0:


(18) $$\left\{ \begin{array}{c} \rho {\left(\eta_{v}\right)}^{\rho -1}Tr\left({\left(\Phi W\right)}^{\text{T}}L_{v}\left(\Phi W\right)\right)-\text{ξ}=0\\ \sum_{v=1}^{V}\eta_{v}-1=0 \end{array}\right.$$

where η_*v*_ can be estimated by:


(19) $$\eta_{v}={\left(\frac{1}{Tr\left({\left(\Phi W\right)}^{\text{T}}L_{v}\left(\Phi W\right)\right)}\right)}^{\frac{1}{\rho -1}}/\sum_{v=1}^{V}{\left(\frac{1}{Tr\left({\left(\Phi W\right)}^{\text{T}}L_{v}\left(\Phi W\right)\right)}\right)}^{\frac{1}{\rho -1}}$$

**FIGURE 2:**
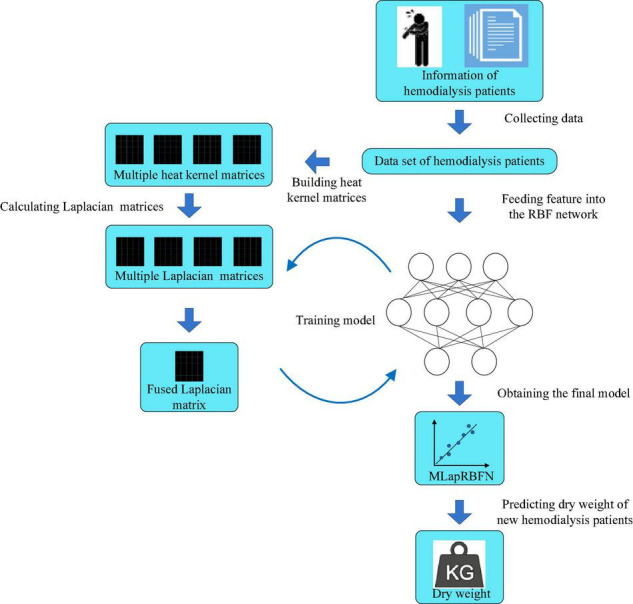
Flow chart of multiple Laplacian-regularized RBF network (MLapRBFN).

The process of MLapRBFN is listed in Algorithm 1 and Figure 2.

**Algorithm 1: Algorithm of multiple Laplacian-regularized RBFN (MLapRBFN).**

**Require:** Training set {*x*_*i*_, *y*_*i*_}, *i* = 1, 2, …, *N*, new samples{*x*_*new*, *j*_}, *j* = 1, 2, …, *M*, the hidden layer nodes (*p*), the iterations *tmax*, parameters of λ_1_and λ_2_;

**Ensure:** The predictive values of $\left\{y_{j}^{te}\right\},j=1,2,\ldots ,M$

(1) Using *k*-means to select *p* centers and width (σ ) for RBF function. Calculating the *W* (training set) and Laplacian matrices *L*_*v*_, *v* = 1, 2, …, *V* by Eqs 1, 10, and 11. Initializing $\eta_{v}^{\left(0\right)}=1/V,v=1,2,\ldots ,V$;

(2) Setting *t* = 0, estimate the initial *W*^(0)^ with Eq. 7;

**Repeat**

(3) Update the *G*^(*t* + 1)^with $G^{\left(t+1\right)}={\left[\begin{array}{ccc} \frac{1}{2{||W_{1}^{\left(t\right)}||}_{2}} & & \\ & \ddots & \\ & & \frac{1}{2{||W_{p}^{\left(t\right)}||}_{2}} \end{array}\right]}_{p\times p}$

(4) Update *W*^(*t* + 1)^ via Eq. 15d;

(5) Update $\eta_{v}^{\left(t+1\right)},v=1,2,\ldots ,V$ via Eq. 19;

(6) Calculating $L^{*\left(t+1\right)}=\sum_{v=1}^{V}\eta_{v}^{\left(t+1\right)}L_{v}$

**until** *t* > *tmax*;

(7) Calculate the output matrix Φ_*new*_(test set);

(8) Predict {*y*_*new*, *j*_}, *j* = 1, 2, …, *M* by $y_{new}^{*}=\Phi_{new}W^{*}$.

## Results and Discussion

In this section, we perform 10-fold cross-validation (10-CV) to test the predictive performance of different models, the BCM device, multiple kernel support vector regression (MKSVR), linear regression (LR), back propagation-based artificial neural network [ANN (BP)], and multi-kernel ridge regression (MKRR).

### Evaluation Measurements

Some traditional assessment methods include correlation coefficient (*R*), *R* square, root mean square error (RMSE), empirical cumulative distribution plot (ECDP), and Bland–Altman analysis. In particular, The Bland–Altman analysis usually can evaluate the agreement between two methods, and determines whether the two methods can be replaced with each other.

### Selection of Optimal Parameters

**FIGURE 3:**
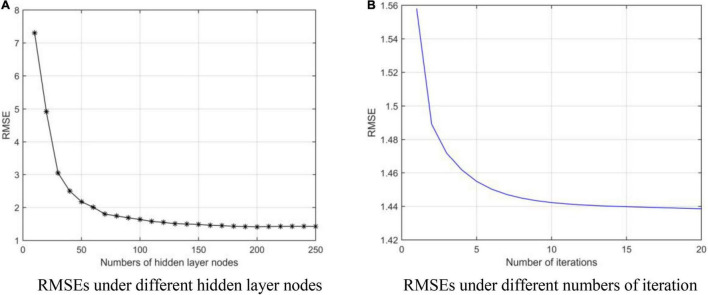
RMSEs under different parameters. (A) Root mean square errors (RMSEs) under different hidden layer nodes. (B) RMSEs under different numbers of iteration.

**FIGURE 4:**
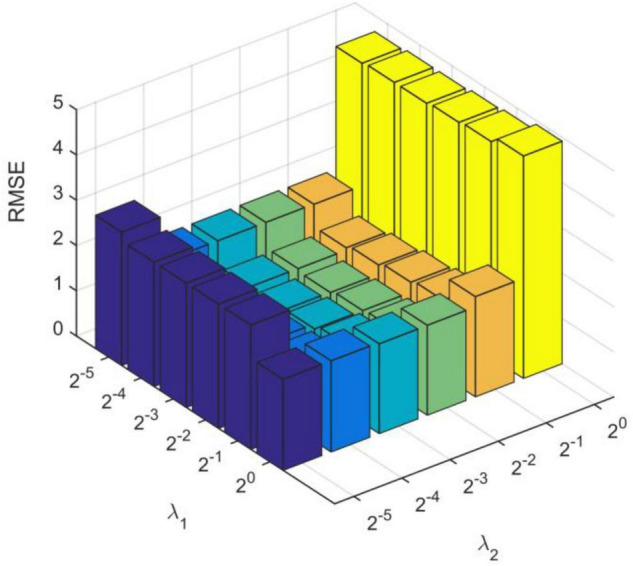
Root mean square errors under different regularization coefficients.

We obtain the optimal model parameters, such as the number of hidden layer nodes (the number of clusters), regularization parameters, and number of optimization iterations. First, we fix the number of iterations (*tmax* = 10) and regularization coefficients (λ_1_ = 2^0^, λ_2_ = 2^0^), and adjust the number of hidden layer neurons. In Figure 3A, we test the number of nodes from 10 to 250 with step of 10. After adding 140 neuron nodes, RMSE tends to be flat. In addition, the RMSE also tends to remain unchanged (minimum) after 10 iterations (Figure 3B). Finally, we get the number of hidden layer nodes as 140 and times of iteration as 10, and adjust the regularization coefficient. The results are shown in Figure 4, and the optimal coefficients are λ1=2-3,λ=22-3.

### Comparison With Other Existing Models

**FIGURE 5:**
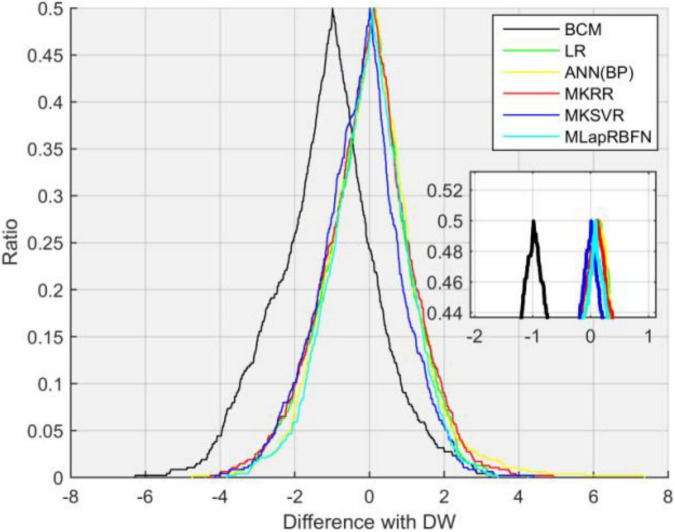
Folded empirical cumulative distribution curves of six methods.

We compare our model with other existing machine learning methods (Guo et al., 2021), which include multi-kernel ridge regression (MKRR), multiple kernel support vector regression (MKSVR), artificial neural network (ANN), linear regression (LR), and BCM measuring instrument. The gold standard is clinical dry weight. In Table 2, our method (MLapRBFN) is better than the ordinary RBF neural network (RBFN) model. The *R* and *R* squared have the highest values of 0.9511 and 0.9432, respectively. In addition, the RMSE of MLapRBFN reaches its lowest value (1.3226). In Table 2 and Figure 5, we can see that our method has the smallest ECDP range (from −3.8174 to 3.4383). The multiple Laplacian regularized RBFN model has multiple graphs for different heat kernels, which contain different information. To effectively integrate each graph of feature into one graph, multi-view learning (MVL) is employed to estimate the weight of each graph. Each graph has different contribution for the model.

**TABLE 2:** Comparison with other methods (10-CV).

Method	*R*	*R* Squared	RMSE	Empirical cumulative distribution plot		
				Highest value	Lowest value	Median value
MKSVR*	0.9412	0.9321	1.3817	4.3962	−4.1273	**0.0082**
MKRR*	0.9399	0.9289	1.5015	4.9227	−4.2604	0.1104
ANN (BP)*	0.9398	0.9295	1.4794	7.3661	−4.7447	0.1324
LR*	0.9403	0.9308	1.4335	4.2524	−.4014	0.1418
BCM*	0.9473	0.9137	1.9694	3.2235	−6.2776	−.9863
RBFN	0.9410	0.9302	1.4514	4.9018	−3.9376	0.0966
MLapRBFN (our method)	**0.9511**	**0.9432**	**1.3226**	3.4383	-3.8174	0.0822

*The results are from previous work of MKSVR. Bold values represents the best performance for each column.

### Bland-Altman Analysis

**FIGURE 6:**
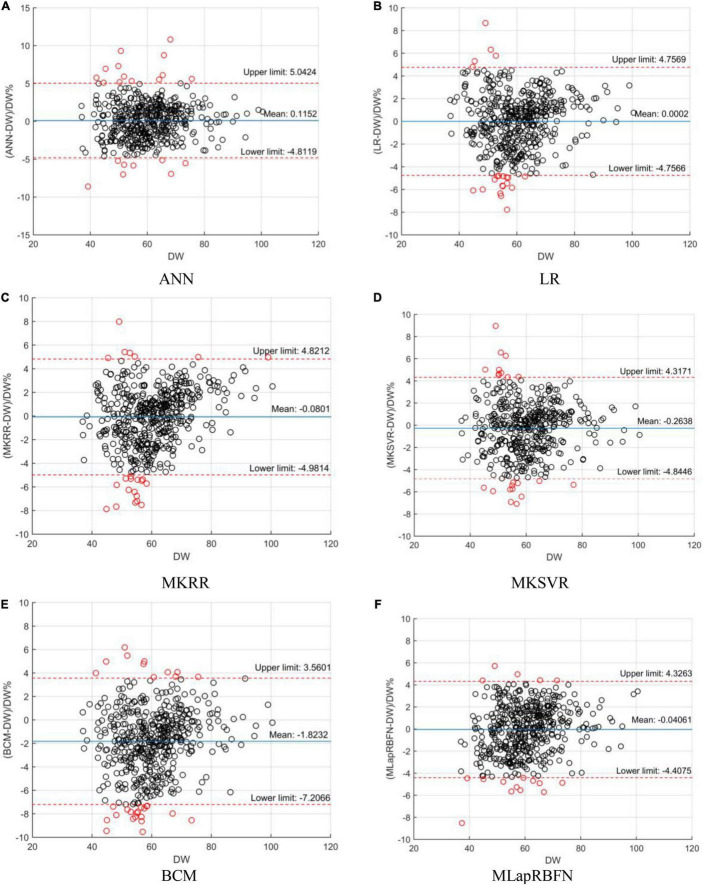
Bland–Altman plot analysis. **(A)** ANN, **(B)** LR, **(C)** MKRR, **(D)** MKSVR, **(E)** BCM, and **(F)** MLapRBFN.

In this section, we employ a Bland–Altman plot to further evaluate the regression error of different models. In Figure 6 and Table 3, several models evaluate the agreement with clinical DW. MLapRBFN achieves the smallest range of -0.2413 to 0.1601 (95% confidence interval). What is more, the number (ratio) of out agreement interval is the key indicator to evaluate whether the two methods are equivalent. For the predictive models, the number (ratio) should be all less than 24 (5%). In Table 3, all the predictive models meet this standard. In particular, our method obtains the least number (ratio) of 17 (3.57%). If 95% of the samples are in agreement range, the predictive models are clinically acceptable. It can be seen that our method can replace clinical methods.

**TABLE 3:** Bland–Altman plot analysis of the models.

Model	Differences with DW (%)			Limits of agreement (%)		
	Mean	SD	95% confidence interval	Lower limit	Upper limit	Number (ratio) of out agreement interval
MKSVR*	−0.2638	2.3372	−0.4743 to -0.05329	−4.8446	4.3171	22/476 (4.62%)
MKRR*	−0.0801	2.5007	−0.3053 to 0.1451	−4.9814	4.8212	23/476 (4.83%)
ANN (BP)*	0.1152	2.5139	−0.1112 to 0.3416	−4.8119	5.0424	22/476 (4.62%)
LR*	0.0002	2.4269	−0.2184 to 0.2187	−4.7566	4.7569	21/476 (4.41%)
BCM*	−1.8232	2.7466	−2.0706 to −1.5759	−7.2066	3.5601	30/476 (6.30%)
MLapRBFN (our method)	−0.04061	2.2280	−0.2413 to 0.1601	−4.4075	4.3263	17/476 (3.57%)

*The results are from previous work of MKSVR (Guo et al., 2021).

## Conclusion

The limitations of BCM and clinical are time-consuming and laborious. In our study, a MLapRBFN method is developed to predict the DW of hemodialysis patients. Different from standard RBFN, our method contains multiple Laplace regularization terms, and uses an efficient iterative algorithm to solve the model. MKRR, LR, MKSVR, and ANN are compared with our model. Bland-Altman analysis and RMSE are the main evaluation methods. In the Bland-Altman analysis of MLapRBFN, the number of out agreement interval is the least (17 samples).

In the fields of medicine (Esteva et al., 2017; Xiao et al., 2017; Hu et al., 2018; Deng et al., 2019; Huang et al., 2020; Zhou et al., 2020; Yang H. et al., 2021), pharmacy (Wang et al., 2020), and biology (Wei et al., 2014, 2017, 2019; Fajila, 2019; Wang Y. et al., 2019; Wang et al., 2021; Yang C. et al., 2021; Zou et al., 2021), artificial intelligence technology has solved lots of predictive tasks. In future studies, more data of hemodialysis patients will be collected, and a deep neural network (Dao et al., 2020a,b; Lv et al., 2020) with stronger representation ability to accurately estimate the DW of hemodialysis patients will be built.

## Data Availability Statement

The original contributions presented in the study are included in the article/supplementary material, further inquiries can be directed to the corresponding authors.

## Ethics Statement

The studies involving human participants were reviewed and approved by the experimental protocol was established and approved by the Human Ethics Committee (Wuxi and Northern Jiangsu People’s Hospital Ethics Committee). The ethical approval numbers are 2018KY-001 and KYLLKS201813. The patients/participants provided their written informed consent to participate in this study.

## Author Contributions

XG and WZ did the experiments and wrote the manuscript. YD, CL, and XG designed the method. YY, YZ, AD, QL, and YC revised the manuscript. All authors have read and approved the final manuscript.

## Conflict of Interest

The authors declare that the research was conducted in the absence of any commercial or financial relationships that could be construed as a potential conflict of interest.

## Publisher’s Note

All claims expressed in this article are solely those of the authors and do not necessarily represent those of their affiliated organizations, or those of the publisher, the editors and the reviewers. Any product that may be evaluated in this article, or claim that may be made by its manufacturer, is not guaranteed or endorsed by the publisher.
